# Supplementary material for: HIVprotI: an integrated web based platform for prediction and design of HIV proteins inhibitors
Source: J Cheminform. 2018 Mar 9;10:12. doi: 10.1186/s13321-018-0266-y (PMC5845081; doi:10.1186/s13321-018-0266-y)
Supplement: Supplementary file 2 — Additional file 2. Source code of HIVProtI web server. [file 13321_2018_266_MOESM2_ESM.zip › HIVprotI_Source-code/hivprotI/weka-3-6-6/doc/weka/estimators/CheckEstimator.EstTypes.html]

CheckEstimator.EstTypes


---


|  |  |  |
| --- | --- | --- |
| **Overview** \| **Package** \| **Class** \| **Tree** \| **Deprecated** \| **Index** \| **Help** \| **Weka's home** | |  |
| **PREV CLASS**   **NEXT CLASS** | **FRAMES**    **NO FRAMES**     **All Classes** |  |
| SUMMARY: NESTED \| FIELD \| CONSTR \| METHOD | DETAIL: FIELD \| CONSTR \| METHOD |  |


---


## weka.estimators Class CheckEstimator.EstTypes

```
java.lang.Object
  weka.estimators.CheckEstimator.EstTypes
```

**All Implemented Interfaces:**: RevisionHandler

**Enclosing class:**: CheckEstimator

---

``` public static class CheckEstimator.EstTypes extends java.lang.Object implements RevisionHandler ```

public class that contains info about the chosen attribute type
estimator work on one attribute only

---

**Constructor Summary**

`CheckEstimator.EstTypes()`
  
          Constructor

`CheckEstimator.EstTypes(boolean i,
boolean w,
boolean s)`
  
          Constructor


| **Method Summary** | |
| --- | --- |
| `java.lang.String` | `getRevision()`             Returns the revision string. |

**Methods inherited from class java.lang.Object**

`equals, getClass, hashCode, notify, notifyAll, toString, wait, wait, wait`

**Constructor Detail**

### CheckEstimator.EstTypes

```
public CheckEstimator.EstTypes()
```

:   Constructor

---


### CheckEstimator.EstTypes

```
public CheckEstimator.EstTypes(boolean i,
                               boolean w,
                               boolean s)
```

:   Constructor


**Method Detail**

### getRevision

```
public java.lang.String getRevision()
```

:   Returns the revision string.

    :   **Specified by:**: `getRevision` in interface `RevisionHandler`
    :   **Returns:**: the revision


---


|  |  |  |
| --- | --- | --- |
| **Overview** \| **Package** \| **Class** \| **Tree** \| **Deprecated** \| **Index** \| **Help** \| **Weka's home** | |  |
| **PREV CLASS**   **NEXT CLASS** | **FRAMES**    **NO FRAMES**     **All Classes** |  |
| SUMMARY: NESTED \| FIELD \| CONSTR \| METHOD | DETAIL: FIELD \| CONSTR \| METHOD |  |


---
